# Neutrophils in non-small cell lung cancer and immunotherapy with PD-1/PD-L1 inhibitors

**DOI:** 10.1186/s12967-025-07084-z

**Published:** 2025-11-18

**Authors:** Shaojie Hu, Chenxi Yan, Yitao Tian, Wei Sun

**Affiliations:** Department of Thoracic Surgery, Tongji Hospital, Tongji Medical College, Huazhong University of Science and Technology, Wuhan, 430030 Hubei China

**Keywords:** Tumor-associated neutrophils, Non-small cell lung cancer, Immunotherapy, Tumorigenesis, Inflammation

## Abstract

Neutrophils, which represent about 50–70% of circulating leukocytes in humans, have been viewed as short-lived effector cells of the innate immune system with a primary role in the clearance of pathogens and taking part in inflammatory processes. More recent evidence shows that neutrophils make up a significant portion of the inflammatory cell infiltration in many types of cancer, including non-small cell lung cancer (NSCLC). These tumor-associated neutrophils (TANs) can be divided into protumorigenic and antitumorigenic phenotypes. The biological features and functions of these TANs are under the influence of the tumor microenvironment. TANs also play a role in determining the responsiveness of NSCLC to immunotherapies targeting the programmed cell death-1 (PD-1)/programmed death ligand 1 (PD-L1) axis. This review aimed to summarize recent findings regarding the role of TANs in NSCLC progression and PD-1/PD-L1 targeted immunotherapy.

## Introduction

Neutrophils are the predominant circulating leukocyte population involving in microbial clearance at sites of infection and in wound healing [1]. The microenvironment of tumors often resembles that of chronic inflammation. Increased numbers of neutrophils have been observed in several tumors [2, 3]. Recent studies show that neutrophils dominate the immune cell composition in non-small cell lung cancer (NSCLC) [4]. Tumor microenvironments (TME) affect lifespan and the function of neutrophils [5]. Tumor-associated neutrophils (TANs) are polarized into anti-tumoral N1 and pro-tumoral N2 TANs subgroups [6]. N1 and N2 TANs are induced by IFN-β and TGF-β, respectively [7]. Although the binary N1/N2 classification is oversimplified the roles of TANs in tumor progression, the overall N1 signature is cytotoxic to tumor cells, whereas N2 neutrophils contribute to tumor growth [8].

The role of TANs in tumor progression is related to the stage of NSCLC. The studies have shown that in the early stage of cancer, most neutrophils recruited into the tumor microenvironment are N1 type and show little inhibitory effect on T cell-mediated responses [9]. The antitumor potential of neutrophils decreases during tumor progression [10]. Neutrophil-derived proteases are involved in regulating the tumor microenvironment. Serine proteases produced by neutrophils cleave cytokines such as TNF-α, IL-2, and IL-6, thereby impairing their functionality [11]. Moreover, several studies have revealed that neutrophil extracellular traps (NETs) formation plays a crucial role in promoting metastasis [12–14].

TANs generate diverse proinflammatory and anti-inflammatory mediators to directly affect tumor growth and influence adaptive immune responses [15]. TANs could promote tumor progression by suppressing immune function. The IL-6/STAT3 pathway is involved in the upregulation of PD-L1 expression on neutrophils [16, 17]. TANs inhibit the proliferation of CD4^+^ T cells and the antitumor ability of NK cells through the PD-1/PD-L1 axis [18, 19]. Thus, remodeling neutrophils by PD-L1 could be a potential strategy to enhance the efficacy of PD-1 blockade-based immunotherapy.

This review is focused on: (1) biologic and functional properties of neutrophils. (2) TANs in NSCLC; and (3) the roles of neutrophils in PD-1/PD-L1 targeted immunotherapy.

### Biologic and functional properties of neutrophils

Neutrophils produced in the bone marrow have a lifespan of fewer than 24 h in circulation [20]. Traditionally, neutrophils are considered to be originated from common myeloid lineage progenitors, situated within the bone marrow and extramedullary tissues, such as the spleen [21]. Recent studies have shown that neutrophils are differentiated from lymphoid-primed multipotent progenitors (LMPPs) [22]. LMPPs differentiate into granulocyte-monocyte progenitor cells (GMPs), which are the myeloid committed progenitor cells. Neutrophils are differentiated from GMPs [23]. The granulocyte colony stimulating factor (G-CSF) is involved in regulating the differentiation process [24]. Bone marrow is also the site for clearance of aged circulating neutrophils [23]. Senescent neutrophils marked increased surface expression of CXCR4 and CCR5 and decreased expression of CXCR2 and CD62L. These phenotypic properties of senescent neutrophils ensure homing back to the bone marrow where aged neutrophils are destroyed by macrophages [23, 25]. The biological significance of aged circulating neutrophils homing back to bone marrow has not been well understood.

Neutrophils are usually collected from the blood by density gradient centrifugation. The classical neutrophils are heavier than Ficoll-Paque, known as ‘normal density neutrophils’(NDNs) or ‘high-density neutrophils’ (HDNs) [26]. Recent studies have identified ‘low-density neutrophils’ (LDNs) in which neutrophils are lighter than Ficoll-Paque [27]. NDNs include both terminally differentiated and immature neutrophils. Some of the immature neutrophils also exist in the LDNs fraction [28]. As neutrophils migrate into inflammatory foci, various chemokines, cytokines, pathogen-associated molecular patterns (PAMPs), and damage-associated molecular patterns (DAMPs) prime and activate the neutrophils [29, 30].

Neutrophils are considered major participants during inflammation, autoimmunity, and cancer. Typically, they are recruited to infection and inflammatory sites very quickly and they have a capacity for eliminating pathogens, particularly bacteria and fungi. Upon activation, neutrophils produce large amount of superoxide anion and ensuring reactive oxygen species to kill phagocytized microbes. NADPH oxidase is responsible for superoxide anion production [31]. In addition, releasing NETs is a typical approach to eliminating extracellular microorganisms. NETs can immobilize pathogens to limit their spread and facilitate the phagocytosis of trapped microorganisms. NETs can also annihilate pathogens through antimicrobial histones and proteases [32].

### Neutrophile heterogeneity and plasticity

Circulating HDNs are mature and cytotoxic, they usually exert antitumor functions. Circulating LDNs include mature cells and myeloid-derived suppressor cells (MDSCs). In humans, HDNs are characterized by high surface expression of CD10, CD11b, CD16, and CD62L, whereas in mice, they are typically identified by high expression of Ly6G and CD11b [33]. LDNs in human are characterized by low CD16 expression, high levels of CD33 and CD66b, and are typically CD10-negative or exhibit low CD10 expression [34]. The LDNs of which mature cells are stem from HDNs in a TGF-β dependent fashion. Compared with HDNs, the mature LDNs have similar N2 neutrophil phenotype but acquire reduced neutrophil functions and protumoral contribution. Immature MDSCs are not cytotoxic, they possess ring-shaped nucleus and immunosuppressive functions related to tumor promotion [35]. MDSCs constitute a heterogeneous population of immature myeloid progenitor cells, which are broadly classified into two principal subsets: polymorphonuclear (PMN)-MDSCs and monocytic (M-MDSCs) groups [36]. The phenotype of PMN-MDSCs is described as CD11b^+^ CD14^−^CD15^+^ CD33^+^ CD66b^+^ HLA-DR^−^Lin^−^, but M-MDSCs is described as CD11b^+^CD14^+^CD15^−^CD33^+^CD66b^+^HLA-DR^−/low^Lin^−^ [37, 38]. No specific marker exists to reliably distinguish immunosuppressive PMN-MDSCs from neutrophils, but PMN-MDSCs express several different markers. Youn et al. distinguished PMN-MDSCs from neutrophils based on their higher expression of CD115 and CD244 and limited phagocytic activity [39]. Subsequently, Condamine et al. established LOX-1 as a potent and specific marker for the identification and isolation of PMN-MDSCs [40].

The migration of neutrophils into tumor tissues is regulated by the concerted action of key mediators, such as IL-17, G-CSF, and ELR^+^ chemokines [41, 42]. TANs in tumor-bearing mice have been conceptually segregated into N1 and N2 subtypes. N1 TANs display a pro-inflammatory and antitumor phenotype, exemplified by high TNF-α and low arginase activity. In contrast, N2 TANs possess a protumorigenic and immunosuppressive profile, characterized by elevated expression of CCL2, CCL3, and high arginase levels [43]. A hallmark of anti-tumorigenic N1 TANs is the production of immunostimulatory factors (TNF-α, CCL3, ICAM-1) and reduced expression of Arginase-1. In contrast, pro-tumorigenic N2 TANs are defined by elevated production of a distinct set of chemokines (CCL2, CCL3, CCL4, CCL8, CCL12, CCL17, CXCL1, CXCL2, CXCL8, CXCL16) [44, 45]. Neutrophils are increasingly recognized for their significant heterogeneity. In the context of cancer, recent studies have revealed several unique neutrophil subpopulations, each resulting from the tumor microenvironment. The functional role of neutrophils within tissues is not fixed but can be reprogrammed based on the specific stimuli (e.g., chemokines) present in the local microenvironment, a process governed by their remarkable plasticity. Genetic ablation of IFN-β promotes the polarization of N2 neutrophils in both murine and human models [46]. This N2 phenotype was associated with diminished tumoricidal capacity, reduced NETosis, and lower expression of ICAM1 and TNF-α. Consequently, these findings indicate that IFN-β is directly involved in polarizing neutrophils towards an N1 state, thereby stimulating their anti-tumor functions. The cytokine IL-35 has been demonstrated to promote N2 neutrophil polarization by upregulating the expression of G-CSF and IL-6, consequently enhancing neutrophil recruitment and infiltration into the tumor microenvironment [47]. Tumor-derived TGF-β is associated with the accumulation of N2-polarized neutrophils and facilitates tumor growth and progression. Indeed, TGF-β blockade resulted in the recruitment of neutrophils with enhanced cytotoxic activity, characterized by elevated expression of TNF-α CCL3, and ICAM-1, alongside reduced arginase-1 levels [44, 48]. Moreover, TGF-β inhibition potentiated an anti-tumor T-cell response, in which neutrophils functioned as critical effector cells. Overall, the IFN-β and TGF-β signaling pathways play pivotal roles in driving the polarization of N1 and N2 neutrophils, respectively.

The phenotypic and functional status of neutrophils is influenced by metabolic factors in the tumor microenvironment. Neutrophils are canonically viewed as cells that are primarily dependent on glycolytic metabolism to fuel their effector functions. Neutrophils isolated from mice bearing early-stage orthotopic lung cancer exhibited upregulation of genes encoding endoplasmic reticulum (ER) stress pathway components, and they showed elevated expression of genes related to energy production and nucleic acid metabolism, accompanied by increased oxidative phosphorylation, higher glycolytic rates, and substantially greater ATP levels than control neutrophils [49]. In a genetically engineered mouse model of lung adenocarcinoma, Ancey et al. demonstrated that TANs showed upregulation of the glucose transporter GLUT1 and underwent a metabolic shift toward enhanced glycolysis, as evidenced by an elevated extracellular acidification rate in response to glucose, increased glucose uptake, and higher ATP production compared to neutrophils from healthy lung [50]. GLUT1 deletion enhanced intratumoral neutrophil turnover, reduced SiglecF expression on TANs, and ultimately attenuated tumor growth while synergizing with radiotherapy to improve treatment outcomes. GLUT1 expression in TANs dictates their role in tumor progression, governing their switch from an anti-tumor to a pro-tumor phenotype. The upregulation of MCT4 and CAIX in lung squamous cell carcinoma (LUSC) suggests that hypoxia and lactate efflux may contribute to an immunosuppressive tumor microenvironment. This likely occurs through mechanisms such as the induction of PD-L1 expression and T-cell anergy under inflammatory conditions—a concept further corroborated by the increased presence of PD-L1^+^ neutrophils [51, 52]. Lipid accumulation in tumor-infiltrating MDSCs is correlated with their immunosuppressive potency. In lung cancer, granulocyte-macrophage colony-stimulating factor (GM-CSF) derived from tumor cells activated STAT5 signaling, which transcriptionally upregulated fatty acid transport protein 2 (FATP2) expression in PMN-MDSCs. Genetic deletion of FATP2 abolished the immunosuppressive function of these cells. Mechanistically, FATP2 facilitated the uptake of arachidonic acid and its subsequent conversion to prostaglandin E2 (PGE2), which was essential for their suppressive activity. In human NSCLC, neutrophils engage Annexin A2 (ANXA2) signals through the TLR2/MYD88 axis, which upregulates arginase 1 (ARG1) expression. This promotes immunosuppression by depleting amino acids and consequently impairing T-cell function and viability [53]. It is worth noting that ARG1 in human is stored in an inactive state within the granules of neutrophils and becomes activated upon release.IL-8 secreted by NSCLC tumor cells promotes the extracellular release of ARG1 from infiltrating neutrophils [54]. The enzyme indoleamine 2,3-dioxygenase (IDO) facilitates immune evasion by tumors and MDSCs through their role in tryptophan depletion, which impairs cytotoxic T cell responses and survival [55]. A key finding was that IDO ablation suppressed AMPK signaling in MDSCs, while paradoxically enhancing it in CD8^+^ T cells. This T cell-intrinsic AMPK activation was associated with a gain of effector function. Changes in lung metabolism can promote the metastasis of extrapulmonary tumors, concurrently inducing functional and phenotypic alterations in neutrophils that may support this process. In the oxygen-rich environment of the lungs, a metabolic reprogramming from glucose to fatty acid utilization is critical for lung metastasis in triple-negative breast cancer. With the shift of metabolism, a distinct proliferative neutrophil subset (Ly6G^+^ApoE^+^Ki67^+^) was found [56]. This specific neutrophil subset co-expressesed the proliferation marker Ki67 along with S100A4 and Thbs1, they were instrumental in sculpting the immunosuppressive landscape of the metastatic niche.

### Interplay between TANs and immune cells

Compared to circulating blood neutrophils, TANs isolated from early-stage human lung cancer exhibited an activated phenotype, characterized by low CD62L and high CD54 expression, along with a distinct chemokine receptor profile including CCR5, CCR7, CXCR3, and CXCR4. These TANs secreted significant levels of MCP-1 and MIP-1α, and TANs enhanced T cell proliferation and interferon-γ (IFN-γ) production. Cross-talk between TANs and activated T cells resulted in pronounced upregulation of the costimulatory molecules CD54, CD86, OX40L, and 4-1BBL on neutrophils, reinforcing T cell expansion through a positive-feedback mechanism [57]. Therefore, in the earliest stages of lung cancer, TANs do not exhibit immunosuppressive activity; instead, they promote anti-tumor immunity by enhancing T cell responses. A high intratumoral neutrophil burden in NSCLC showed a markedly suppressed T-cell response, evidenced by reduced expression of cytotoxic T-cell markers (CD8A, CD8B, GZMA, GZMB), lower infiltration of CD3^+^CD8^+^ T cells, and downregulation of IFN-γ-associated genes [58]. In a study by Xu et al., NETs formation was found to be elevated in patients with lung adenocarcinoma (LUAD). These NETs promoted LUAD progression by impairing CD8^+^ T cell cytotoxicity—specifically through the suppression of perforin, granzyme A, and granzyme B expression. This effect was mediated via YTHDF2-dependent degradation of SLC2A3 mRNA [59]. P2RX1-deficient neutrophils upregulated PD-L1 expression by inducing fatty acid metabolism, thereby suppressing T cell proliferation and granzyme B production while promoting T cell exhaustion—collectively contributing to immunosuppression in NSCLC [60]. Neutrophils serve as a major source of uridine phosphorylase-1 (UPP1) in metastatic cancer. UPP1-expressing neutrophils exhibit upregulated adhesion molecules that impair their motility in the pre-metastatic lung and inhibit T-cell proliferation [61]. Additionally, uracil—the catalytic product of UPP1—mediates enhanced fibronectin deposition within the lung extracellular matrix. Tumor-associated neutrophils isolated from a murine lung cancer model promoted immunosuppression by robustly inducing CD8^+^ T cell apoptosis through the TNFα pathway, a process that involves nitric oxide (NO) production [62]. PPM1D/Wip1 functions as a negative regulator of the tumor suppressor p53, and mutations in PPM1D within immune cells are associated with poorer clinical outcomes in lung cancer. PPM1d-deficient neutrophils exhibit enhanced tumor infiltration and potently suppress lung cancer growth [63]. Inhibition of Wip1 in both human and mouse neutrophils enhances neutrophile anti-tumor phenotypes and the proliferation of co-cultured cytotoxic T cells, which demonstrates that Wip1 inhibition in neutrophils potentiates antitumor immune responses [63]. MDSCs play a critical role in promoting tumor progression by orchestrating immunosuppressive mechanisms within the tumor microenvironment [64]. MDSCs exhibit elevated nitric oxide production via enhanced arginase 1 expression, resulting in L-arginine depletion and subsequent T cell cycle arrest [65]. Additionally, MDSCs downregulate T cell receptor expression, further contributing to T cell anergy. Active MDSCs upregulate PD-L1 expression, the engagement of PD-L1 on MDSCs with PD-1 on T cells induces T cell exhaustion [66, 67].

Following stimulation with lipopolysaccharide plus IL-2 or IL-15/IL-18, neutrophils directly interact with and enhance the activity of NK cells. Neutrophils directly provide co-stimulatory signals to NK cells through CD18/ICAM-3 interaction, thereby inducing the production of IFN-γ [68]. Neutrophils release soluble factors such as IL-1β and IL-18 to recruit and activate NK cells, which subsequently exhibit enhanced cytotoxic activity and cytokine production. NK cells conditioned by neutrophils promoted the maturation of dendritic cells, which in turn enhanced T cell proliferation and IFN-γ production [69]. Reactive oxygen species (ROS) produced via neutrophil-derived myeloperoxidase (MPO) can suppress the antitumor activity of NK cells. Neutrophils mediate B cell chemotaxis through the secretion of TNF-α, particularly following exposure to chemokines such as CXCL12 or CXCL13 [70]. Neutrophils secrete B-cell-activating factor via a G-CSF-dependent mechanism, thereby promoting the accelerated differentiation of plasma cells [71]. Neutrophils directly interacted with dendritic cells (DCs), enhancing TNF-α expression and promoting DCs activation [72]. Co-culture with human neutrophils promoted the upregulation of membrane CD86 and HLA-DR on DCs through a cell contact-dependent mechanism, thus eliciting antigen-specific T cell responses [73]. TANs highly expressed CCL17 and CCL2, which recruit regulatory T cells (Tregs) and macrophages, respectively, thereby contributing to the impairment of CD8^+^ T cell function [74].

### Tumor-associated neutrophils in NSCLC

#### Genetic and phenotypic characteristics of TANs in lung cancer

The roles of neutrophils in NSCLC have been extensively investigated in recent years. The canonical neutrophil markers CD45^+^CD11b^+^CD15^hi^CD66b^+^MPO^hi^Arg-1^+^IL-5Rα^−^ are expressed in TANs and neutrophils in peripheral blood (PBNs) [75]. Compared with PBNs in lung carcinomas patients, increased levels of CCR5, CCR7, CXCR3, and CXCR4 are expressed in TANs, whereas CXCR1 and CXCR2 expression are downregulated [76]. Besides the phenotypic alterations in TANs, the tumor microenvironment also changes the lifespan of recruited short-lived circulating neutrophils. In the tumor microenvironment, TANs exhibit prolonged survival than circulating neutrophils [57]. This effect is closely related to pro-inflammatory factors, such as IFN-γ, IL-6, IL-8, and GM-CSF [77, 78]. Those pro-inflammatory factors prolong the survival of TANs via restricting their apoptosis.

To delineate the cell type-specific transcriptomic landscape of cancer cells and the tumor microenvironment in advanced NSCLC, Wu et al. profiled 42 tissue biopsy samples from patients with stage III/IV NSCLC using single-cell RNA sequencing (scRNA-seq) [79]. It was demonstrated that cancer cells exhibited elevated expression of the ligands CXCL1, CXCL2, CXCL3, and CXCL8, which mediate signaling through the receptors CXCR1 and CXCR2 on TANs. Neutrophil infiltration is more pronounced in human LUSC than in LUAD, a difference attributed to distinct tumor microenvironment characteristics [80]. The transcription factor SOX2—a lineage-specific oncogene in squamous cell carcinomas—was shown to be overexpressed and to facilitate the accumulation of TANs through the upregulation of CXCL5 expression [81]. Hao et al. identified TANs within the tumor region of LUSC, characterized by expression of CXCR2, CSF3R, and CXCL8. These TANs exhibited upregulated expression of interleukin-1 receptor antagonist (IL1RN), suggesting a potential mechanism of immunosuppression mediated through IL1RN [82]. Employing scRNA-seq, Salcher et al. constructed a detailed atlas that reveals the heterogeneous landscape and plasticity of TANs in NSCLC [83]. Their analysis revealed that TANs can be categorized into four distinct subsets based on their gene expression profiles. Among these, TAN-1 exhibits high expression of interleukin-1 receptor antagonist, a recognized marker of activated neutrophils that negatively regulates IL-8 secretion to restrain excessive neutrophilic inflammatory activity. The TAN-2 subcluster was defined by prominent expression of major histocompatibility complex class II genes—including HLA-DRA, CD74, HLA-DMB, and HLA-DRB1, which suggesting a phenotype with potential antigen-presenting capability. A defining feature of the TAN-3 subcluster was the elevated expression of key proinflammatory cytokines (C15orf48, CCL3, CCL4, CSTB) and galectin-3. The TAN-4 subcluster exhibited elevated expression of ribosomal genes, including RPS12, RPL3, RPN2, and RPL23. The prevailing hypothesis suggests that TAN transcriptomes arise from the reprogramming of neutrophils already resident within the tumor microenvironment, rather than from the recruitment of circulating neutrophils [84]. Recent scRNA-seq studies of circulating neutrophils in cancer patients revealed no significant transcriptomic reprogramming during circulation [85, 86]. However, Lattanzi et al. performed scRNA-seq experiments on circulating NSCLC mature PMN-MDSCs and identified a prominent scRNA-seq cluster that was distinctly enriched in immunosuppressive and pro-tumor transcriptomic features [87]. Compared with TANs in NSCLC, Lattanzi et al. found that a circulating scRNA-seq cluster shared several common genes and transcription factor regulons associated with hypoxia response, angiogenesis, and metabolic reprogramming. Therefore, a subset of circulating neutrophils might undergo a degree of transcriptomic reprogramming prior to infiltrating the tumor microenvironment in NSCLC.

In a mouse model of lung adenocarcinoma, tumors harboring a *KRAS*^*G12C*^ mutation exhibited pronounced neutrophil aggregation and concomitant exclusion of tumor-infiltrating lymphocytes. Treatment with the *KRAS*^*G12C*^ inhibitor MRTX1257 significantly attenuated neutrophil accumulation and enhanced T-cell infiltration, suggesting a remodeling of the immunosuppressive tumor microenvironment [88, 89]. Through single-cell RNA sequencing, Ye et al. identified a significantly enriched neutrophil population within the tumor microenvironment of lung adenocarcinoma patients harboring co-occurring *KRAS* and *TP53* mutations, suggesting a potential role of neutrophil infiltration in the immune contexture of this molecular subtype [90]. Intercellular communication analysis revealed that neutrophils within the tumor microenvironment harboring *KRAS* and *TP53* mutations exhibited significantly enhanced crosstalk with endothelial cells, B cells, macrophages, and epithelial cells compared to those in wild-type settings, particularly through ligand-receptor signaling axes involving oncostatin M, calcitonin receptor, and interleukin 1. Inactivating aberrations in *STK11* have been identified as critical drivers of an immunosuppressive tumor microenvironment in NSCLC, characterized by diminished infiltration of CD3^+^, CD4^+^, and CD8^+^ tumor-infiltrating lymphocytes and an accumulation of TANs [91]. Transcriptomic and post-transcriptomic analyses revealed a marked reduction in a pronounced neutrophil degranulation signature [92]. Genetic loss of *STK11* remodels the tumor cytokine milieu, inducing upregulated expression of proinflammatory mediators—including CXCL7, G-CSF, IL-1β, and IL-6—which promotes neutrophil recruitment and suppresses T-effector cell function [93]. Compared to tumors with *KRAS* mutation alone, those harboring concurrent *KRAS* and *STK11* mutations exhibited increased infiltration of TANs, which demonstrated elevated expression of immunosuppressive markers including arginase 1 and interleukin-10 [93]. In addition, loss of *STK11* function has been demonstrated to promote robust immune escape in murine lung tumor models through the recruitment of CD11b^+^Gr-1^+^TANs and a concomitant reduction in T cell infiltration, thereby fostering an tumor immunosuppressive microenvironment [94]. Mutations in *KEAP1* significantly dysregulate the tumor immune microenvironment in lung adenocarcinoma, contributing to an immunosuppressive phenotype. LUAD patients with *KEAP1* mutations exhibited significantly elevated neutrophil infiltration and experienced inferior clinical outcomes compared to those with wild-type *KEAP1* [95]. The elevated neutrophil infiltration observed in LUAD patients with *KEAP1* mutations was associated with concurrent upregulation of enhanced activity of the nitrogen metabolism signaling pathway.

#### Effects and targets of TANs in NSCLC

Anti-tumoral N1 phenotype and pro-tumoral N2 phenotype are induced by IFN-β and TGF-β respectively. Some researchers found that tumor metastasis could be restricted by the deletion of TGF-β, which is related to the reduction of TGF-β1, iNOS, and arginase 1 [96]. As anti-tumor agents, interferons are widely used in clinical trials. IFN-β induces neutrophil phenotype change into antitumor TANs in humans. Lacking IFN-β could result in protumor neutrophils dominating lung tumors [46]. The conversion of neutrophils from tumor-promoting to tumor-suppressing is dependent on NK cells activation. Thus, targeting NK cells may be a potential approach to treating cancer via regulating the balance between N1 and N2 neutrophils [18].

**Fig. 1:**
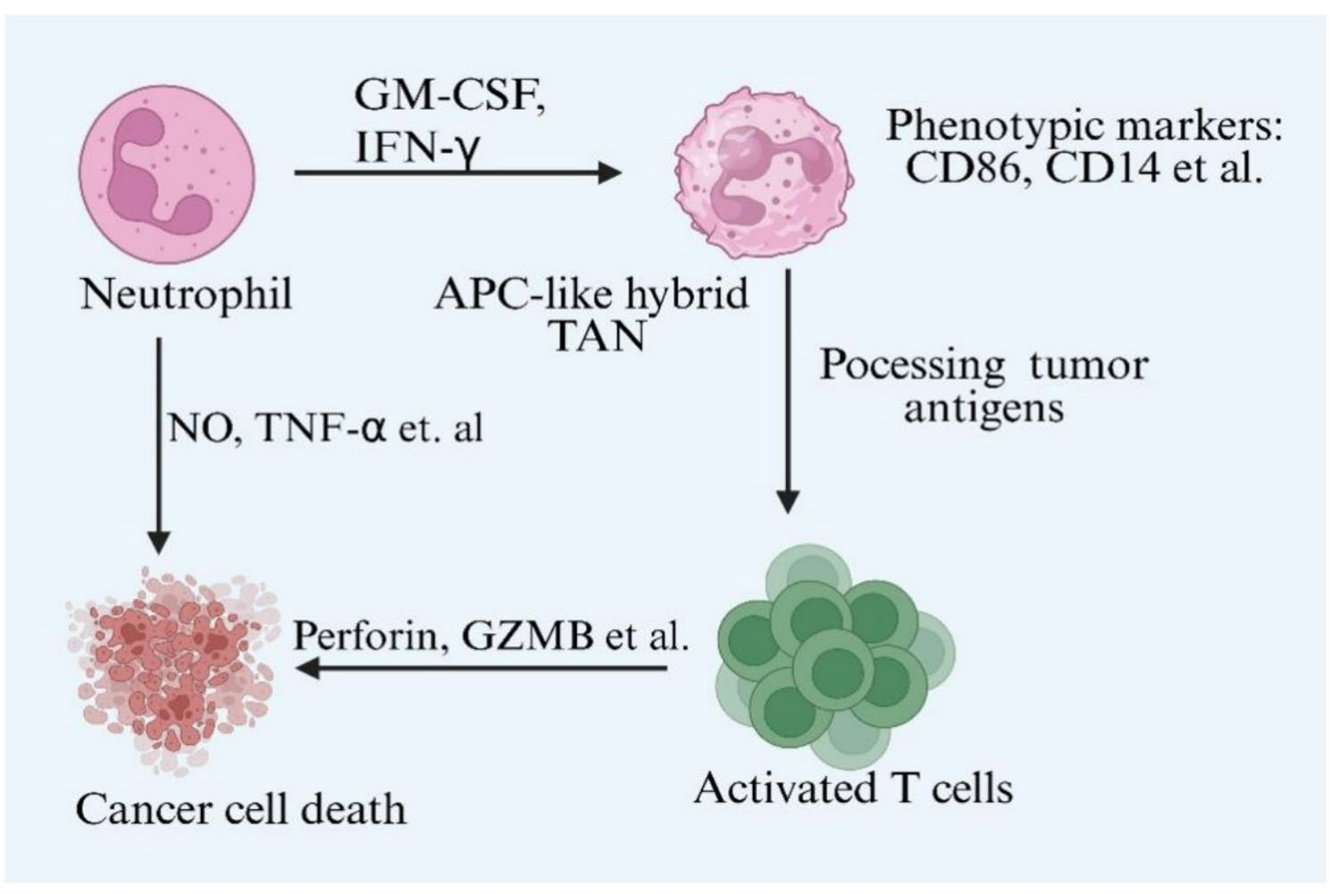
In the early stages of non-small cell lung cancer, neutrophils can exert anti-tumor effects. During this early phase, neutrophils can directly kill tumor cells by releasing cytotoxic molecules, such as nitric oxide and tumor necrosis factor-alpha. Furthermore, under the influence of interferon-gamma and granulocyte–macrophage colony-stimulating factor, neutrophils can differentiate into a subset possessing antigen-presenting capabilities. This specific neutrophil subset can present tumor antigens to T cells, thereby contributing to the suppression of early-stage lung cancer progression. Created with Biorender.com

In the early stage of NSCLC, neutrophils recruited into the tumor microenvironment are predominately N1 type. Mishalian and colleagues have shown that neutrophils from early tumors display potent cytotoxic effects on tumor cells through increased production of TNF-α, NO, and H_2_O_2_ [97]. Hybrid TANs are observed in tumors smaller than 3 cm. Hybrid TANs exhibit markers of professional antigen-presenting cells (APCs), such as HLA-DR and HLA-ABC, and also express canonical TANs phenotypic markers (CD15, CD66b, CD11b) under the influence of IFN-γ and GM-CSF [98, 99]. The APC-like hybrid TANs can take up, degrade, and process tumor antigens via high-affinity IgG receptors FcγRI and FcγRII [100, 101]. The early-stage lung tumor microenvironment may promote the differentiation of APC-like hybrid TANs and enhance anti-tumor effects by activating T-cell mediated antitumor responses. Interestingly, neutrophils infiltration in late-stage tumors (5–7 cm in diameter) only display the canonical phenotype of TANs [99]. Neutrophil-mediated anti-tumor mechanisms during the initial phase of NSCLC are show in Fig. 1.

Several mechanisms underlying the decline in the antitumor potential of neutrophils during tumor progression have been reported [30]. Neutrophils released serine proteases promote tumor progression by degradation of tissues and facilitating tumor invasion [102]. Neutrophils can synthesize cyclooxygenase (COX)-derived prostanoid PGE2 and the 5-lipoxygenase-dependent leukotrienes [103]. COX-2 exerts a significant role in lung cancer pathogenesis and promotes survival of lung adenocarcinoma cells in vitro through releasing PGE2 [104].

Growing research has shown that cancer incidence worldwide is due to infectious agents [105]. Recently Nejman et al. analyzed the tumor microbiome in NSCLC of current smokers and never-smokers [106]. The aberrant microbiota in the lung are relevant to COPD, and most lung cancer patients coexist with COPD [107]. Nontypeable Haemophilus influenzae (NTHi) is the main bacterial pathogen found in COPD patients [108]. The colonization of NTHi causes chronic neutrophilic inflammation and significantly accelerates lung tumor growth [109]. IL-17C cytokine produced by epithelial cells is involved in regulating the tumor-promoting effect induced by NTHi in a murine model [110]. The extrinsic inflammation-induced recruitment of neutrophils releases serine proteases leading to degradation of antitumorigenic factor thrombospondin-1 (Tsp-1). Elimination of those proteases reduces Tsp-1 degradation and restricts lung metastasis [111]. Inflammatory neutrophils contribute to lung cancer metastasis could be due to regulating the neutrophil protease–Tsp-1 axis. In addition, neutrophil infiltration could act on blood vessels leading to tumor hypoxia [112]. Increased tumor hypoxia stabilizes Snail expression in lung tumor cells mediated by hypoxia inducible factor-1α (HIF-1α). Snail is a vital epithelial-to-mesenchymal transition (EMT) transcription factor and facilitates cancer progression on several immune cell populations [113]. Interestingly, increased Snail expression in lung tumor cells enhances the recruitment of neutrophils into tumors. Snail-expressing tumor cells increase the secretion of CXCL2 which is the ligand of CXCR2, promoting the recruitment of neutrophils [112]. This positive feedback loop of neutrophil recruitment and Snail expression accelerate lung cancer progression.

Neutrophils utilize the ability to counteract an infection through phagocytosis and the release of NETs. NETs are neutrophil-derived extruded DNA webs and antimicrobial proteins [114]. NETs are released from neutrophils in response to various stimuli, such as G-CSF stimulation [115]. Though NETs are characterized as an anti-microbial mechanism, NETs are closely associated with cancer progression. Cools-Lartigue et al. utilized a murine model transplanted with A549 cells to explore the role of NETs in NSCLC [116]. Their studies have shown that NETs deposited in microvascular and trapped circulating A549 cells within DNA webs. The NETs trapping of A549 cells induces the formation of hepatic micro metastases. Various stimuli can trigger NETs formation, including receptor-mediated signals or through calcium channel formation. NETs are composed of extracellular structures such as DNA, histones, and neutrophil granules containing neutrophil elastase (NE) and MPO [117]. α1-antitrypsin is a protease inhibitor targeting NE in particular [118]. The imbalance between α1-antitrypsin and NE is an underlying cause of lung tissue damage that may create a favorable host environment for carcinogenesis [119]. Imbalance of α1-antitrypsin and NE is also considered to be an important factor to promote tumor growth [120]. Hattar and colleagues have shown that coculture of A549 cells with neutrophils isolated from human peripheral blood causes an obvious A549 cell proliferation which is mediated by NE [121]. The NE degrades insulin receptor substrate-1 (IRS-1) in the endosomal compartment of tumor cells, which causes enhanced interaction between phosphatidylinositol 3-kinase (PI3K) and the potent mitogen platelet-derived growth factor receptor (PDGFR), and consequently, the PI3K axis facilitates lung tumor cells proliferation [122]. In addition, NE can degradate elastic fiber thereby forming intense deposition of elastotic material including newly formed elastokines in lung cancer [123]. The disruption of the elastic fibers might facilitate lung cancer cell dissemination especially stromal invasion [124]. Elastokines can accelerate the progression of NSCLC via inducing IL-8 production and causing neutrophil recruitment [125]. NE has been reported to induce tumor cell dissemination and migration in vivo. The release of NE from activated neutrophils stimulates protease activating receptor 2 (PAR2) in A549 cells, which causes the activation of EGFR via HB-EGF [126]. Sustained over-activation of EGFR in lung cancer increases the expression of EMT-inducing factors thereby favoring lung cancer metastasis [127, 128]. Human neutrophil elastase, a well-established mediator in chronic obstructive pulmonary disease, has recently been implicated in the progression of NSCLC. TANs contribute to tumor angiogenesis through the secretion of pro-angiogenic factors such as VEGF and IL-8, as well as proteases including matrix metalloproteinases (MMPs) and elastases [30, 129, 130]. In the extracellular space, NE generates elastin fragments—termed morphoelastokines—that potently promote cancer cell invasiveness and angiogenesis [125]. Reduced VEGF expression and decreased density of CD31^+^ endothelial cells in NE-deficient *KRAS* mutant mice, suggesting that neutrophil NE promotes tumor angiogenesis [131]. NE mediates the proteolytic cleavage of VEGF, generating diffusible fragments that enhance the recruitment and activation of pro-inflammatory immune cells via the VEGFR1/Akt signaling pathway, which associated with significant upregulation in tumor cell proliferation and angiogenesis in lung cancer [131, 132]. NAD(P)H:quinone oxidoreductase 1 (NQO1) upregulates peptidyl-prolyl cis–trans isomerase A (PPIA). PPIA engages CD147 on neutrophils, triggering the release of NETs and neutrophil elastase, which in turn promote tumor progression, invasiveness, and pulmonary colonization. The NQO1–PPIA–CD147–NETs signaling axis plays a critical role in mediating breast cancer lung metastasis by orchestrating neutrophil activation and extracellular trap release [133]. Chronic stress induces remodeling of the pulmonary microenvironment and disrupts the normal circadian rhythm of neutrophils, leading to enhanced formation of NETs and expression of CXCL2 and CXCL5 via glucocorticoid-mediated mechanisms [134]. Chronic stress induced neutrophil NETs formation and fosters a metastasis-promoting microenvironment in the lung via glucocorticoid-mediated mechanisms. Neutrophils actively induced tumour necrosis which is associated with poor prognosis in cancer. A tumor-elicited neutrophil population characterized by a Ly6G^High^Ly6C^Low^phenotype exhibited impaired extravasation in response to inflammatory stimuli but demonstrated an enhanced capacity to form NETs [135]. Neutrophils and NETs occluded the tumor vasculature, thereby inducing hypoxia and necrosis in downstream vascular beds. Cancer cells proximal to these necrotic foci underwent EMT, which in turn enhanced their metastatic potential and increased the burden of lung metastasis. Tumor cell-released autophagosomes elicited the formation of NETs in both human and murine neutrophils through the involvement of high mobility group box 1 and subsequent activation of the TLR4–Myd88–ERK/p38 signaling axis. The NETs-mediated suppression of T-cell function in vitro and in vivo played a pivotal role in fostering immunosuppression in the lung premetastatic niche [136].

**Fig. 2:**
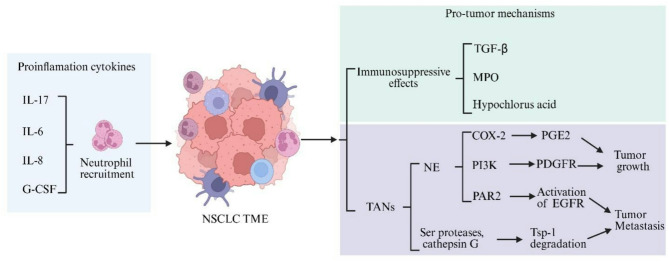
During the advanced stages of non-small cell lung cancer, neutrophils predominantly exhibit pro-tumorigenic functions. As NSCLC progresses, the anti-tumor effects of neutrophils diminish, while their pro-tumor activities become dominant. Stimulated by various cytokines, increased numbers of neutrophils are recruited into the tumor microenvironment. These neutrophils foster an immunosuppressive milieu through the release of transforming growth factor-beta, myeloperoxidase, and hypochlorous acid, which suppresses T cell-mediated antitumor immunity. Moreover, neutrophils promote tumor growth and metastasis by releasing neutrophil elastase and serine proteases. Created with Biorender.com

MPO catalyzes the formation of reactive oxygen intermediates, including hypochlorous acid (HOCl) [137]. The MPO/HOCl system plays an important role in microbial killing by neutrophils. Several epidemiological studies have shown that a polymorphism (-463G → A transition) in the promoter region of the MPO gene decreases the risk of developing lung cancer [138, 139]. A large amount of HOCl formation is linked to the induction of DNA damage and inhibits the activity of DNA damage repair and that may cause mutagenesis and carcinogenesis [140, 141]. Neutrophils infiltrated in tumors express increased level of MPO and have been demonstrated to inhibit the function of CD4^+^ and CD8^+^ T cells [142]. It appears MPO plays an important role in promoting tumor growth. MPO is significantly associated with recurrence of NSCLC after surgical resection, chemotherapy or radiation therapy [143]. N-acetyl lysyl tyrosyl cysteine amide (KYC), a tripeptide inhibitor of MPO, could significantly reduce lewis lung carcinoma growth in mice [144]. The pro-oncogenic effects of MPO in lung cancer, namely enhanced cellular proliferation and reduced apoptosis, were mediated by the activation of the AKT and ERK signaling pathways via increased phosphorylation [143]. Pharmacological inhibition of MPO with 4-aminobenzoic acid hydrazide was sufficient to reduce the lung cancer burden in mice, as evidenced by a significant decrease in tumor progression. Other MPO inhibitors (e.g., AZD3241, AZD4831, SNT-8370, UFM24) have been investigated for their anti-inflammatory effects in neutrophilic lung injury, but their roles in lung cancer treatment warrant further exploration [145–147]. Neutrophil-driven tumor promotion mechanisms during NSCLC progression are summarized in Fig. 2.

Given the marked heterogeneity and functional plasticity of neutrophils—which have been demonstrated to exert both pro-tumor and anti-tumor activities in NSCLC—therapeutic strategies aimed at modulating neutrophil phenotypes and functions hold promise for suppressing tumor progression. Neutrophil elastase promotes tumor progression and metastasis in NSCLC through mechanisms such as extracellular matrix degradation and immune modulation. Consequently, targeting NE has emerged as a promising therapeutic strategy for NSCLC treatment. NE inhibitors, such as Sivelestat, GW311616A, and AZD9668, have been investigated as potential therapeutic agents for the treatment of lung cancer [148–150]. Inada et al. have utilized ONO-5046xNa, a specific neutrophil elastase inhibitor, to investigate its effects on the growth of NSCLC [151]. Their results demonstrate that daily intraperitoneal injection of ONO-5046xNa suppresses NSCLC growth. NETs suppressed T-cell function and facilitated the establishment of an immunosuppressive microenvironment within the lung premetastatic niche. DNase I not only suppresses the formation and function of NETs, but its combination with αPD-L1 also restored T-cell function and abrogated tumor metastasis in lung [136]. CXCR2 inhibitors, including SX-682(NCT03161431), represent an emerging therapeutic strategy aimed at disrupting neutrophil-driven immunosuppression in NSCLC [152]. By targeting the CXCR2 signaling axis, these inhibitors aim to restore anti-tumor immunity and overcome resistance to existing therapies. Mice bearing lung tumors that received combined treatment with anti-PD1 and SX-682 recruited more lymphocytes infiltrating the malignant tumor mass with the induction of interferon-γ responsive genes. Thus neutrophil antagonism with CXCR2 inhibitor could potentially serve as a therapeutic approach for improving the therapeutic efficacy of immune checkpoint inhibitor treatments in lung cancer [153].

### Neutrophils in immunotherapy with PD-1/PD-L1 inhibitors

PD-1/PD-L1 axis blockade-based immunotherapy has shown promising and durable responses in some patients with NSCLC. Currently, one of the crucial issues regarding PD-1 based immunotherapies is to identify patients who respond to PD-1 inhibitors. Neutrophil to lymphocyte ratio (NLR) is an index of systemic inflammation and relevant to prognosis in several cancers such as NSCLC [154]. In advanced NSCLC patients who treated with PD-1 blockade, Zer and colleagues have demonstrated that the baseline NLR ≤ 4 was closely related to better disease control rate, lasting treatment duration, and overall survival compared to patients with baseline NLR > 4 [155]. For patients with metastatic NSCLC receiving immunotherapy, those with a NLR ≥ 4 exhibited significantly shorter median progression-free survival (PFS) [156]. Although NLR values are dynamic, accumulating evidence supports an inverse correlation between lower NLR values and enhanced therapeutic efficacy of immunotherapy in lung cancer patients [157, 158]. Due to prognostic value of NLR in advanced NSCLC, the researchers thought NLR had potential predictive value for advanced NSCLC control and response during PD-1 axis inhibitors treatment. Therefore, the NLR may serve as an important biomarker in clinical practice for predicting the efficacy of immunotherapy. The optimal NLR cutoff values vary across different types of cancer [159]. As discussed previously, there are substantial differences in gene expression profiles and phenotypic characteristics between peripheral blood neutrophils and tumor-infiltrating neutrophils. Peripheral blood neutrophils may not accurately reflect the status of neutrophils within the tumor microenvironment. Consequently, the reliability of NLR as a predictive biomarker is somewhat questionable, and relying solely on this simplistic and crude metric may introduce bias. Thus, NLR should only be considered as a supplementary reference in clinical decision-making and not be used as a standalone indicator.

Defensins promote various host immune responses against microorganisms by having chemotactic activity for host immune cells including dendritic antigen-presenting cells and T lymphocytes, by inducing the production of cytokines such as interleukin-8 by epithelial cells, by degranulating mast cells, and by enhancing in vivo immunological reactions [160]. Berghmans and colleagues utilized matrix-assisted laser desorption/ionization mass spectrometry imaging (MALDI -MSI) to investigate potential relationship of neutrophil defensin expression with PD-1 immunotherapy response [161]. MALDI -MSI is a multiplexed analysis grasping the lung tumor microenvironment on molecular level [162]. By applying MALDI- MSI method to pretreatment biopsies of advanced NSCLC patients responding and nonresponding to PD-1 immunotherapy, they found that an increased level of neutrophil defensin 1, 2 and 3 expression is related to a positive immunotherapy response, suggesting that neutrophil defensins could serve a biomarker to predict the response to immunotherapy [161]. In order to understand further the function of defensins in this study, the authors cocultured NSCLC cell line with PBMC in the presence of neutrophil defensins. The results show that an increased secretion of IFN-γ was observed after treatment with neutrophil defensins in the coculture. This indicated that neutrophil defensins might activate the immune response and explained the role of the neutrophil defensins in anti-PD-1 immunotherapy response [161, 163].

*STK11/LKB1* is an inactivated tumor suppressor in NSCLC, especially in tumors harboring *KRAS* mutations [164]. Ablation of *STK11/LKB1* in a *KRAS*-driven lung cancer mouse model has led to the recruitment and accumulation of neutrophils to suppress T-cell function [93]. Koyama et al. have shown that *LKB1* inactivation significantly increases the expression of proinflammatory cytokines, such as IL1α, CXCL7, G-CSF, and IL-6 in lung cancer cells. These cytokines are associated with the recruitment of neutrophils, leading to increased recruitment and accumulation of neutrophils to suppress T-cell function [93, 165]. Elevation of IL1α activated IL6–STAT3 signaling pathway together with IL6 caused neutrophil accumulation in *Kras/Lkb1* tumor microenvironment [166]. Moreover, *Lkb1* inactivation reduces the expression of PD-L1 in lung cancer cells, PD-1–targeting therapy is not effective in treating *Lkb1*-deficient lung tumors [167]. Utilizing antibodies against Ly-6G/Gr-1 and IL-6 depleting TANs results in remarkably improving the number and functions of T cells in the *Kras/Lkb1* model [93]. Tumor-derived IL-6 activates the STAT3–ERK1/2 signaling cascade in neutrophils, thereby promoting their prolonged survival and functional activation [168]. Activated neutrophils contribute to tumor progression not only by promoting cancer cell metastasis and endothelial angiogenesis, but also by inducing the differentiation of cancer-associated fibroblasts. IL-6 synergizes with G-CSF to modulate neutrophil development and function, resulting in the reprogramming of signaling pathway responsiveness—most notably enhancing STAT3 activation [169]. Enhanced STAT3 activation in TANs leads to significantly reduced secretion of myeloperoxidase, neutrophil elastase, and TRAIL, while concurrently inducing markedly elevated expression of MMP9 and Bv8 genes, which favors tumor angiogenesis and growth. Patel et al. reported that IL-6 upregulation was a key mediator of immunosuppression in EGFR-TKI-resistant NSCLC. Blockade of IL-6 not only restored antitumor immunity but also overcame resistance to anti-PD-1 therapy, and this synergistic effect provides a strong mechanistic foundation for subsequent combinatorial clinical investigations [170]. Elevated circulating IL-6 levels were identified as an independent unfavorable prognostic factor for both progression-free survival and overall survival in patients with NSCLC treated with immune checkpoint blockade [171]. Evidence suggests that combined blockade of IL-6 and PD-1 can restore CD8^+^ T cell effector function, thereby enhancing antitumor immunity and suppressing tumor growth. This synergistic approach represents a promising clinical strategy for the treatment of NSCLC [172]. Beyond the cytokine IL-6, IL-8 plays a critical and non-redundant role in the tumor microenvironment by promoting the growth and recruitment of neutrophils. Elevated circulating levels of IL-8 exhibit considerable predictive accuracy for identifying subsets of individuals who are unlikely to derive clinical benefit from checkpoint inhibitor-based immunotherapy [173]. IL-8 functions as a potent chemoattractant for neutrophils through its interaction with the cognate receptors CXCR1 and CXCR2. IL-8 mediates neutrophil chemotaxis and induces the formation and release of NETs, which contributes to cancer development via facilitating escape from cytotoxic immune cells [174]. Lung cancer cell lines can secrete high levels of IL-8, and IL-8 significantly reduced apoptosis in neutrophils, increased PD-L1 expression in neutrophils in vitro, thereby affecting tumor progression [175]. Therefore, the blockade of IL-8 or its cognate receptors (CXCR1 and CXCR2), when combined with anti-PD-L1 checkpoint inhibitors, represents a significant strategy for enhancing therapeutic efficacy [176]. Tang et al. discovered that elevated IL-8 levels within the lung cancer TME enhance neutrophil infiltration and concurrently induce the differentiation of a CD74^high^SiglecF^low^ neutrophil subset, endowing it with antigen-presenting capacity [177]. This CD74^high^SiglecF^low^ neutrophil subpopulation activates tumor-specific T cells to exert anti-tumor effects, ultimately leading to significant suppression of NSCLC progression. Knockdown of CD74 in neutrophils inhibits T cell activation, whereas CD74 agonists markedly enhance T cell activation. Consequently, the high expression of CD74 on neutrophils presents a promising therapeutic target for augmenting lung cancer immunotherapy. IL-17A is reportedly involved in lung cancer inflammation and PD-1 checkpoint blockade therapy [178]. Akbay et al. found IL-17A was involved in lung cancer inflammation and PD-1 checkpoint blockade therapy [178]. Their study indicated an increase in circulating levels of IL-17A in patients with lung adenocarcinoma. IL17A recruits and activates neutrophils in the tumor immune microenvironment by inducing the production of IL-6 in their research. They thought IL-17A and recruited neutrophils cause lung cancer inflammation and shape the lung cancer immune microenvironment where T cells function are suppressed, leading to the resistance to PD-1 immune checkpoint blockade. As previously described, tumor-derived TGF-β promotes the accumulation of N2-polarized neutrophils and contributes to tumor growth and malignant progression. Inhibition of TGF-β promoted the recruitment of tumor-infiltrating neutrophils exhibiting potent cytotoxic capabilities and a tumor-suppressive N1-like phenotype [44, 48]. Therefore, therapeutic inhibition at multiple nodal points of the TGF-β signaling pathway represents a promising strategy for developing novel combinatorial regimens with immune checkpoint inhibitors in the treatment of non-small cell lung cancer. M7824, a novel bifunctional fusion protein concurrently targeting PD-L1 and trapping TGF-β, ameliorated TGF-β1-induced immunosuppression in both in vitro and in vivo models of NSCLC [179]. M7824 was observed to have manageable safety and associated with notable immunologic changes, exhibited promising antitumor activity as a second-line therapy in patients with NSCLC, according to results from a phase I, open-label clinical trial (NCT02517398) [180]. Other several novel bifunctional agents, including SHR-1701 and YM101, have been developed to counteract resistance to PD-1/PD-L1 inhibitors in lung cancer through simultaneous blockade of both PD-1/PD-L1 and TGF-β pathways [181]. CXCL5 facilitates neutrophil recruitment into the tumor microenvironment, where they impair CD8⁺T cell-dependent antitumor immunity. CXCL5 induced the expression of PDL1 in neutrophils, these PD-L1^+^neutrophils further exacerbate CD8^+^ T cell exhaustion following lung cancer establishment. Importantly, combined therapy using anti-CXCL5 and anti-PD-L1 antibodies synergistically suppresses tumor growth in vivo [182, 183]. CXCL5 mediates the recruitment of MDSCs by binding to its cognate receptor, CXCR2, expressed on the surface of these immunosuppressive cells. Notably, pharmacological inhibition of CXCR2 signaling has been shown to effectively enhance the therapeutic efficacy of lung cancer immunotherapy [184]. The CXCR2 antagonist SX-682 synergized with immune checkpoint inhibitors by inhibiting the tumor infiltration of G-MDSCs, thereby enhancing anti-tumor immunity [185]. Combination therapy with anti-PD-1 and anti-CTLA-4 antibodies induces a robust initial antitumor efficacy, accompanied by evidence of immune activation; however, extended treatment results in acquired resistance, marked by the expansion of immunosuppressive populations such as regulatory T cells and neutrophils. The addition of an anti-Ly6G blocking antibody to anti-PD-1/CTLA-4 therapy effectively reversed treatment resistance and restored CD8⁺T cell activity in lung cancer models, by targeting Ly6G on neutrophils [186].

**Fig. 3:**
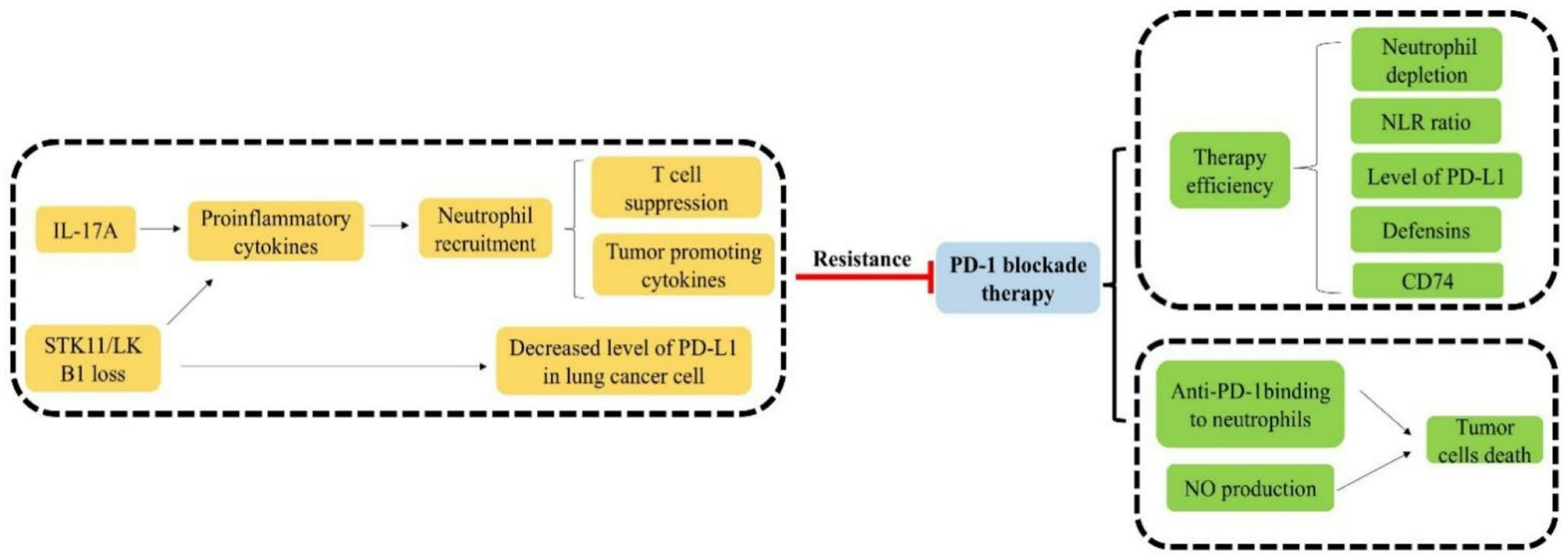
Neutrophils play complex yet critical roles in immunotherapy for non-small cell lung cancer. They serve as predictive biomarkers for immunotherapy efficacy, with low neutrophil-to-lymphocyte ratio, elevated expression of neutrophil defensins (e.g., HNP1-3), and CD74 expression correlating with positive outcomes. Therapeutically, blockade of PD-1 on neutrophils can enhance their anti-tumor activity. However, neutrophils also contribute to immunotherapy resistance. This is notably mediated by IL-6, which drives massive neutrophil infiltration into the tumor microenvironment under conditions of *STK11/LKB1* deficiency or IL-17A stimulation. These tumor-associated neutrophils suppress T cell-mediated anti-tumor immunity, ultimately leading to immunotherapy failure

Recent study using a murine patient-derived xenograft (PDX) model of early-stage NSCLC has investigated the tumor cells and immune non-lymphoid cells in responses to PD-1 inhibitor treatment [187]. In this study, the authors utilized NOD-SCID gamma mice which are characterized by lack of T and B lymphocytes as well as NK cell functionality. NSCLC tumor model treated with anti-PD-1 appeared a fluid with a serous appearance which was confirmed a cellular exudate including acute inflammatory reaction. Interestingly, this situation was only observed in the anti-PD-1 group. The cellular exudate contained of large number of polymorphonuclear leukocytes specifically neutrophils displaying the N1 (anti-tumoral) phenotype. N1 phenotype neutrophils exert anti-tumor function via neutrophil oxidant pathway, specifically NO which synthesized by neutrophil nitric oxide synthase (NOS) [188–190]. NO was detected observably in anti-PD-1 group of this study, indicating neutrophils cause tumor cell death by cytotoxicity and NO production thereby forming nitrotyrosine to induce oxidative damage. Neutrophils in the necrotic areas of tumors are active in anti-PD-1 groups. The necrotic areas of the tumor under the anti-PD-1 treatment differ from those produced by cisplatin, which indicates that the different cell death mechanisms exist. Cisplatin induces tumor cell death through apoptosis [191], but no apoptosis phenomenon appears in the anti-PD-1 antibody treatment group. In order to identify whether anti-PD-1 binds with neutrophils, Martín-Ruiz et.al applied double immunostaining to analyze anti-PD-1 binding sites [187]. They observed that anti-PD1 bound to both FcγR and PD-1 receptors located in the cell membrane surface neutrophils. Aforementioned studies indicated the effector action of neutrophils in anti-PD-1 treatment can be exerted through several processes, including NO production, the binding of anti-PD-1 to PD-1 receptors, and the binding of the anti-PD-1 antibody to FcγR on neutrophil surface. Therefore, neutrophils might act as PD-1 inhibitor effector cells through potential cytotoxic action. However, no precedent studies for direct activation of neutrophil by anti-PD-1 monoclonal antibody. PD-1 and PD-L1 can be expressed on the surface of neutrophils, activation of PD-1 signaling in neutrophil is associated with inhibitory signals of Tregs [192, 193]. Until now there has been rare studies related to PD1 expressed on the surface of neutrophil. Actually the exact role of neutrophil in lung tumor environment is controversial. Faget et al. held the view that neutrophil depletion could revert immune exclusion in NSCLC antiPD-1 treatment [112]. Their results indicated neutrophil depletion via anti-Ly6G or anti-Gr1 antibody normalized blood vessel and regulated immune reprograming through pericytes and endothelial cells, which reverted immune exclusion and PD1 treatment resistant [112, 194, 195]. We summarize the roles of neutrophil-mediated immunotherapy regulation in NSCLC in Fig. 3. Due to the controversial role of neutrophil, more researches are needed to elaborate the relationship between neutrophils and anti-PD-1 immunotherapy clearly.

### Clinical translation and perspective of neutrophils in NSCLC

Peripheral blood contains abundant neutrophils. In clinical practice, peripheral blood sampling for laboratory testing is a simple, convenient, and inexpensive medical procedure. The NLR can predict prognosis across multiple cancer types, although its specific threshold values are not constant [159]. Furthermore, the NLR value may be used to predict the efficacy of immunotherapy in lung cancer, thereby helping to identify suitable candidates for such treatment in advance. However, it is noteworthy that more research is needed on the NLR—particularly regarding specific neutrophil subpopulations and their functional roles. Circulating levels of IL-6 and IL-8 may predict response to immunotherapy in lung cancer patients, potentially through their influence on neutrophil activity. For instance, IL-6 can promote the pro-tumor functions of neutrophils via STAT3 signaling, while IL-8 may induce NETs formation, thereby facilitating tumor metastasis [169, 174].Nevertheless, the precise concentration ranges of circulating IL-6 and IL-8 that predict immunotherapeutic outcomes require further delineation. Moreover, the predictive value of these cytokines must be validated repeatedly across multiple time points, taking into account measurements taken before, during, and after treatment, as well as the number of treatment cycles. Additionally, peripheral blood profiles can be influenced by bacterial and other infections, pathogen-associated molecular patterns and damage-associated molecular patterns can prime and activate the neutrophils, and activated neutrophils can release superoxide anion and NETs to eliminate pathogens [31, 32]. Therefore, such variables could introduce significant confounding effects, potentially obscuring the interpretation of neutrophil-associated biomarker data.

Given the phenotypic and functional plasticity of neutrophils, they can be influenced by the tumor microenvironment to exert either anti-tumor or pro-tumor functions. Consequently, certain neutrophil subpopulations have the potential to serve as biomarkers for predicting the efficacy of cancer therapy. As previously mentioned, tumor-infiltrating neutrophils can be categorized into anti-tumor N1 and pro-tumor N2 subtypes. N1 neutrophils are characterized by high expression of TNF-α, CCL3, and ICAM-1, but low expression of Arginase-1 [44]. Therefore, detecting the expression of these molecules in tumor-associated neutrophils can reflect whether the immune microenvironment is immunosuppressed, which in turn may predict responses to immunotherapy. In the lung cancer microenvironment, IL-18 can induce the differentiation of a CD74^high^SiglecF^low^ neutrophil subset capable of antigen presentation, thereby activating tumor-infiltrating T cells [177]. Reduced CD74 expression on neutrophils significantly impairs the anti-tumor function of T cells, whereas high CD74 expression promotes an anti-tumor immune microenvironment and may synergize with immunotherapy to enhance tumor suppression. Thus, CD74 expression in tumor-infiltrating neutrophils could serve as a predictive biomarker for immunotherapy response. Additionally, neutrophil-derived defensins can stimulate T lymphocytes and induce the production of interferon-gamma, exerting anti-tumor effects [161]. Higher levels of defensin expression in the tumor microenvironment may therefore predict better outcomes following immunotherapy in lung cancer. In the same context, CXCL5 signaling via CXCR2 can upregulate PDL1 expression on neutrophils [182]. High PDL1 expression on neutrophils may lead to interaction with PD-1 on effector CD8^+^ T cells, resulting in T cell exhaustion and impaired anti-tumor activity. Although this induces an immunosuppressive microenvironment, neutrophil PDL1 expression could potentially serve as a predictor of response to immunotherapy. Thus, it would be valuable to collect tumor samples before and after treatment and correlate PDL1 expression levels with clinical outcomes to establish a scoring system. Moreover, neutrophils infiltrating the lung tumor microenvironment express high levels of MPO, which suppresses the anti-tumor functions of both CD4^+^ and CD8^+^ T cells and promotes tumor progression [142]. Therefore, MPO expression may reflect the immunosuppressive nature of the microenvironment. Similarly, NE and NETs released by intratumoral neutrophils can impair T cell function and promote metastasis in non-small cell lung cancer by remodeling the tumor stroma [136, 150]. Inhibiting NE/NETs formation may thus synergize with immune checkpoint inhibitors. In summary, various neutrophil subtypes and their expressed molecules—such as CD74, defensins, PDL1, MPO, NE, and NETs—hold promise as predictive biomarkers for immunotherapy in lung cancer. However, it is unlikely that a single marker will suffice for accurate prediction; instead, a combination of indicators may be necessary. Notably, research progress on neutrophils has lagged behind that of other immune cells, such as T cells and macrophages, largely due to biological challenges including their short lifespan, high plasticity, and phenotypic instability under experimental conditions. These properties also make neutrophils prone to activation and alteration during isolation and manipulation, potentially introducing artifacts. Therefore, methodological rigor is essential—careful attention must be paid to sample processing time, storage conditions, and the selection of appropriate detection techniques such as immunohistochemistry and flow cytometry, depending on the research objectives.

In the context of neutrophil-targeted therapeutic strategies for lung cancer, several inhibitors have been developed, including the CXCR2 inhibitor SX-682, neutrophil elastase inhibitors such as Sivelestat, GW311616A, and AZD9668, as well as the NETs inhibitor DNase I, some of which are currently under clinical investigation [136, 150, 153]. Certain of these agents demonstrate synergistic antitumor effects when combined with immunotherapy. However, it is crucial to select lung cancer patients for immunotherapy based on neutrophil-specific biomarkers to maximize clinical benefit. Unfortunately, clinical trials exploring such strategies remain scarce, primarily due to an insufficient understanding of neutrophil biology. For example, lung cancer patients with comorbid COPD are at significantly increased risk of developing immune-related pneumonia during immunotherapy, wherein neutrophils play a key role [196]. Neutrophil-derived serine proteases, MPO, MMP-8, and MMP-9 contribute to airway extracellular matrix remodeling, thereby promoting COPD pathogenesis [197]. The COPD immune microenvironment is already enriched with neutrophils, and immunotherapy can further amplify pulmonary neutrophil infiltration, potentially exacerbating lung injury [198]. These findings highlight the impact of individual neutrophil profiles on treatment outcomes in lung cancer. Nevertheless, current clinical guidelines do not incorporate neutrophil functional or phenotypic states into therapeutic decision-making, relying instead on routine blood counts mainly to detect infections or other systemic conditions. To achieve true precision medicine, it is essential to account for the functional and phenotypic heterogeneity of neutrophils in individual patients—an approach that will likely become feasible in the near future.

## Conclusions

The presence of neutrophils in the lung tumor microenvironment plays a crucial role in determining the outcome of the host anti-tumor response as well as tumors responding to PD-1 based immunotherapies. TANs are heterogeneous and exert diverse functions at different stages of lung cancer. A more precise classification of TANs to identify their concrete functions is necessary. When and how the antitumor status of the neutrophils will change are still unknown. A simple neutrophil count may not predict the accurate clinical prognosis of lung cancer patients. Therefore, further investigation on neutrophils in NSCLC is required and that could facilitate the selection of potential targets from neutrophils perspective for effective treatment of NSCLC.

## Data Availability

Not applicable.

## Abbreviations

ANXA2: Annexin A2
APC: Antigen-presenting cell
ARG1: Arginase 1
COX: Cyclooxygenase
DAMPs: Damage-associated molecular patterns
DCs: Dendritic cells
EMT: Epithelial-to-mesenchymal transition
ER: Endoplasmic reticulum
FATP2: Fatty acid transport protein 2
G-CSF: Granulocyte colony stimulating factor
GM-CSF: Granulocyte–macrophage colony-stimulating factor
GMPs: Granulocyte-monocyte progenitor cells
HDNs: High-density neutrophils
HIF-1α: Hypoxia inducible factor-1α
HOCl: Hypochlorous acid
IDO: Indoleamine 2,3-dioxygenase
IFN-γ: Interferon-γ
IL1RN: Interleukin-1 receptor antagonist
IRS-1: Insulin receptor substrate-1
LDNs: Low-density neutrophils
LMPPs: Lymphoid-primed multipotent progenitors
LUAD: Lung adenocarcinoma
LUSC: Lung squamous cell carcinoma
MALDI-MSI: Matrix-assisted laser desorption/ionization mass spectrometry imaging
MDSCs: Myeloid-derived suppressor cells
M-MDSCs: Monocytic myeloid-derived suppressor cells
MMPs: Matrix metalloproteinases
MPO: Myeloperoxidase
NDNs: Normal density neutrophils
NE: Neutrophil elastase
NETs: Neutrophil extracellular traps
NLR: Neutrophil to lymphocyte ratio
NO: Nitric oxide
NOS: Nitric oxide synthase
NQO1: NAD(P)H:quinone oxidoreductase 1
NSCLC: Non-small cell lung cancer
NTHi: Noticeable Haemophilus influenzae
PAMPs: Pathogen-associated molecular patterns
PAR2: Protease activating receptor 2
PBNs: Peripheral blood neutrophils
PD-1: Programmed cell death-1
PDGFR: Platelet-derived growth factor receptor
PD-L1: Programmed death ligand 1
PDX: Patient-derived xenograft
PFS: Progression-free survival
PGE2: Prostaglandin E2
PI3K: Phosphatidylinositol 3-kinase
PMN-MDSCs: Polymorphonuclear myeloid-derived suppressor cells
PPIA: Peptidyl-prolyl cis–trans isomerase A
ROS: Reactive oxygen species
scRNA-seq: Single-cell RNA sequencing
TANs: Tumor-associated neutrophils
TME: Tumor microenvironment
Tregs: Regulatory T cells
Tsp-1: Thrombospondin-1
UPP1: Uridine phosphorylase-1
